# High consciousness—low application: sustainable development and sustainable healthcare in undergraduate physiotherapy education in Sweden

**DOI:** 10.3389/fpubh.2024.1509997

**Published:** 2024-12-16

**Authors:** Emma Swärdh, Nina Brodin, Annie Palstam, Anna Pettersson

**Affiliations:** ^1^Division of Physiotherapy, Department of Neurobiology, Care Sciences and Society, Karolinska Institutet, Huddinge, Sweden; ^2^Division of Physiotherapy, Department of Orthopaedics, Danderyd Hospital, Stockholm, Sweden; ^3^School of Health and Welfare, Dalarna University, Falun, Sweden; ^4^Department of Clinical Neuroscience, Institute of Neuroscience and Physiology, Sahlgrenska Academy, University of Gothenburg, Gothenburg, Sweden

**Keywords:** survey, educators, higher education, new ecological paradigm scale, sustainability consciousness questionnaire, sustainability attitudes

## Abstract

**Background:**

Swedish undergraduate physiotherapy education lacks comprehensive integration of sustainable development in curricula. Factors related to educators’ perspectives in preparing future physiotherapists for sustainable development and sustainable healthcare may shed light on this shortcoming.

**Aim:**

This study aims to describe Swedish physiotherapy educators’ (i) consciousness of sustainable development and its inclusion in teaching and learning activities, (ii) ecological worldviews, (iii) attitudes toward sustainability and climate change in physiotherapy, (iv) perceptions of education for sustainable development and sustainable healthcare and (v) examine the relationship between ecological worldview and attitudes toward sustainability and climate change in physiotherapy.

**Method:**

A cross-sectional, descriptive study was performed using a digital survey to collect data from educators within undergraduate physiotherapy education at five higher education institutions in Sweden. Data was collected using the Sustainability Consciousness Questionnaire, the New Ecological Paradigm Scale, the Sustainability Attitudes in Nursing Survey 2, and questions related to knowledge, attitudes, and self-efficacy for education for sustainable development and sustainable healthcare.

**Result:**

Most, but not all, of the 72 educators, (76%) were aware of Agenda 2030 and the sustainable development goals, and 17% included perspectives related to sustainable development in teaching and learning activities. The educators endorsed an eco-centered ecological worldview and had largely positive overall attitudes toward sustainability and climate change within physiotherapy. However, almost one-third (28%) disagreed that issues about climate change should be included in the physiotherapy curriculum. Most agreed about having content knowledge on climate and health (81%), while a smaller part agreed on having pedagogical content knowledge regarding how to inspire or educate for sustainable development (17–28%). There was also a wide variation in perceived self-efficacy in education for sustainable development and sustainable healthcare.

**Conclusion:**

Despite the endorsement of eco-centered ecological worldviews and a rather high consciousness of sustainable development as an overall concept, there remains a disconnect to educational attitudes and actions among Swedish physiotherapy educators. This points to the need to explore the narrative of sustainable development within physiotherapy in Sweden rooted in broader concept understanding, ethics, and reflective practice for sustainable development. A key priority should be to offer new perspectives on professional identity and continuing professional development within sustainable development.

## Introduction

Sustainable development is essential for the well-being of both current society and future generations. To address the global challenges and foster resilient, thriving societies, the United Nations’ Agenda 2030 and its sustainable development goals underscore the interconnectedness of economic growth, social inclusion, and environmental stewardship (1–3). Further, the nested dependency model of sustainable development (4) highlights the environment as the fundamental base supporting human society and economic systems. The concept of planetary boundaries (5) reinforces the environmental limits within which human development must operate. Consistent with this perspective, it is crucial to emphasize that environmental sustainability issues have become an urgent global health concern (6–10). The adverse health effects associated with negative exposure to environmental determinants of health place increasingly high demands on health care (11). At the same time, healthcare services in itself have a role in further contributing to negative health effects by generating emissions that lead to climate change and other environmental impacts such as air-and water pollution (12). While healthcare providers, including physiotherapists, are often less prepared to identify or respond to sustainability issues including environmental threats (13–15), the rising generation of health professionals is increasingly expecting their education to prepare them for building a sustainable future (16, 17). Accordingly, the undergraduate education for the next generation of physiotherapists must be significantly shaped by this concern and prepare students to work for and within a sustainable healthcare system.

International educational agreements urge universities to adopt sustainable practices across teaching and learning and have formulated policies focused on education for sustainable development, particularly the United Nations Decade of Education for Sustainable Development (18). These directives emphasize that education for sustainable development should be integrated into all university programs regardless of academic discipline rather than being limited to sustainability-focused courses. Within health care, the International Association for Health Professions Education, in 2021, provided a consensus statement (19) including a vision for educating a healthcare workforce that can deliver sustainable healthcare and promote planetary health, i.e., that our health depends on our environment. Sustainable healthcare refers to a comprehensive approach to health services that optimally balances the delivery of high-quality care, efficient resource use, and minimal environmental impact while ensuring long-term economic and social viability (20). Three overarching learning outcomes for education for sustainable healthcare have been suggested; “describe how the environment and human health interact at different levels, demonstrate the knowledge and skills needed to improve the environmental sustainability of health systems, and discuss how the duty of a health care professional to protect and promote health is shaped by the dependence of human health on the local and global environment” (21).

Several calls to action for the physiotherapy profession and physiotherapy education to advocate for sustainable health care and planetary health have also been put forth by researchers from primarily the global north, highlighting the need for new ways to think and practice physiotherapy (22–25). Physiotherapy certainly plays a significant role in sustainable healthcare practices by using non-pharmacological and non-invasive treatments that empower patients to manage their own care in promotion, prevention, and rehabilitation (26, 27). Thus, physiotherapy can reduce the need for other more resource-intensive care solutions (24). However, physiotherapy also must reduce its carbon footprint through improvements in clinical practice by reducing waste, recycling, and promoting socially just and eco-friendly interventions for health behaviors such as zero-emission transport and a healthy diet within planetary boundaries in relevant contexts (28, 29), as well as focusing on evidence-based practice and lean service delivery. In addition, physiotherapists can also be involved in the preservation and renewal of ecosystems by engaging in the creation of public health initiatives for nature-based health (30). Physiotherapy educators therefore need to strive to integrate environmental sustainability into undergraduate physiotherapy curricula.

This study is situated within the context of Sweden, a country in the Global North where healthcare accounts for 4.5 percent of the national carbon footprint (31). The discourse on sustainable development in Sweden, much like in many other countries, has been shaped largely by Western paradigms that prioritize economic growth, environmental protection, and social equity (32). These pillars often focus on balancing the three dimensions of sustainable development rather than recognizing the interdependence and potential limits of growth. The governance of higher education in Sweden reflects an interplay between strong state regulation with a civic mission, institutional autonomy, and broader neoliberal trends (33). Academic freedom is explicitly protected by law within research while there is no equivalent explicit legal protection in teaching (34). Since 2006, the Swedish Higher Education Act has stated that higher education should promote sustainability in its activities (35). However, in 2017, the Swedish Higher Education Authority which monitors compliance with laws and regulations found that six of eight universities providing undergraduate physiotherapy education require further development in their work on sustainable development within education (36). Since then, most universities in Sweden have become signatories to the Climate Framework (37), committing to ambitious climate action and integrating sustainability into all aspects of their operations. Thus, this legal and institutional framework reflects Sweden’s broader commitment to sustainable development. Still, up to now, only 3% of all learning outcomes are sustainability-focused in Swedish undergraduate physiotherapy education, when explored through the lens of including all three dimensions of sustainable development (38).

For the successful implementation of both education for sustainable development and education for sustainable healthcare into undergraduate physiotherapy education, it is crucial for educators to comprehensively understand and embrace their role in preparing students for a sustainable future. This level of understanding necessitates that educators recognize sustainable development as a concept, as well as education for sustainable development and sustainable healthcare, but also know how to incorporate it into their teaching and learning activities. While some studies have shown that educators and healthcare professionals are not always sufficiently equipped to tackle sustainability challenges (39–42), a study on Swedish nurses and physicians has shown that they tend to endorse a rather eco-centric perspective in their ecological worldview (43). People with this perspective see humans as part of a broader ecological system, rather than as separate or dominant over nature (44), and are also more likely to engage in individual behaviors that help protect and sustain the environment (45). So far, we lack an understanding of how this translates to health profession educators, particularly educators within physiotherapy.

The aim of this study is therefore to describe Swedish physiotherapy educators’ (i) consciousness of sustainable development and inclusion of sustainable development in teaching and learning activities, (ii) ecological worldviews, (iii) attitudes toward sustainability and climate change in physiotherapy, and (iv) knowledge, attitudes and self-efficacy in education for sustainable development and sustainable healthcare, as well as to (v) examine the relationship between ecological worldview and attitudes toward sustainability and climate change in physiotherapy.

## Materials and methods

### Research design and context

This cross-sectional, descriptive study focused on educators in undergraduate physiotherapy programs in a Swedish higher education context. As part of the adaption to the Bologna Process, the Swedish national study program in physiotherapy at first cycle level comprises 180 European Credit Transfer System credits or 3 years of full-time studies and is offered by eight different higher education institutions across Sweden. All programs adhere to the Qualification descriptor for Bachelor of Science in Physiotherapy (46). In addition to campus-based education, clinical practice is an important part of the education. Clinical practice is an educational experience under the supervision of a qualified physiotherapist in a clinical setting. For example, this could be inpatient care, primary care, or various forms of accommodation and treatment homes within municipal activities. The students in Sweden will complete approximately 1,000 clinical practice hours of a total of 4,800 educational hours during their education. Educators within undergraduate physiotherapy education in Sweden consist mostly of physiotherapists and other professionals such as physicians, psychologists, physiologists, and behavioral scientists. Most educators have their primary employment at a university but some within healthcare. In Sweden, the proportion of women among employed physiotherapists is about 75 percent (47). Of the total number of employees at Swedish higher education institutions with research and teaching duties, irrespective of profession, 47 percent are women and 53 percent are men (48).

### Participants and data collection

Five out of eight higher education institutions providing undergraduate physiotherapy education in Sweden accepted the invitation to participate in the study (Karolinska Institutet, Linköping University, Lund University, Mälardalen University, and Uppsala University). Data were collected from educators teaching or supervising, part-time or full-time, in a campus-based or clinical practice course in an undergraduate physiotherapy program in Sweden. The program directors, or equivalent persons in charge of each undergraduate physiotherapy program sent out information about the study and an invitation to participate to a total of 201 campus-based educators. Coordinators for educators in clinical practice were approached at all five programs, and three of these programs sent out the invitation to participate. Details on the exact number of educators in clinical practice who received the invitation could not be obtained due to logistic problems with duplicates of invitations from different coordinators and a lack of information on reach. Data was collected anonymously through a digital survey consisting of self-reported questions. The educators were provided with a link to the survey through the invitation letter, and it took approximately 10–15 min to complete. Three reminders were sent out to the campus-based educators, but none to the ones within clinical practice. Data was collected from March to May 2023.

### Measures

Demographic characteristics included participants’ sex, age, higher education institution, primary employment, profession, working title/position, participation in the program, and years of teaching/supervising. Other data was collected using other measures: the Sustainability Consciousness Questionnaire–short version (49); ecological worldviews including the New Ecological Paradigm Scale (44), attitudes toward sustainability and climate change in physiotherapy including the adapted Sustainability Attitudes in Nursing Survey 2 (50), and questions of education for sustainable development and sustainable healthcare.

#### Consciousness of sustainable development

Consciousness of sustainable development included knowledge of Agenda 2030, the sustainable development goals, the inclusion of sustainable development content in teaching and learning activities, attendance in courses/seminars on sustainable development in the past year and was collected through questions specifically designed for the present study as well as the Sustainability Consciousness Questionnaire (SCQ-S). The SCQ-S is a valid and reliable measure developed to assess consciousness regarding sustainable development (49). The SCQ-S includes 27 items within three domains: economic, social, and environmental, and further assesses consciousness toward these domains in terms of three psychological factors: knowingness (recognition of the importance of sustainability), attitude (the attitudes toward sustainability), and behavior (the willingness to act toward a sustainable future). The items are rated on 5-point Likert scales, (1 = totally disagree to totally agree =5, with a neutral option in the middle), with higher scores indicating high sustainability consciousness (min 1-max 5). The total SCQ-S score for the psychological factors within each domain is divided by the number of items to give a mean total SCQ-S score for each domain (min 1-max 5). The researchers have permission to use the Swedish version of SCQ-S in this study from the rightsholders (49). The item interrelatedness (Cronbach’s *α*) of the measure was 0.845 for the total score.

#### Ecological worldviews

The revised New Ecological Paradigm Scale (NEP) is a widely used, valid, and reliable measure developed to assess people’s beliefs and attitudes toward ecological issues, and their views on the balance between human activities and the natural world, i.e., ecological worldview (44). The NEP is constructed of 15 items around five aspects, namely; ‘human domination over nature, human exceptionalism, the balance of nature, the risk of an eco-crisis and limits to growth’. The items are rated on 5-point Likert scales. Agreement with items with odd numbers and disagreement with the even-numbered items indicate pro-environmental responses, i.e., the scale for even-numbered questions: 1 = strongly agree, 2 = mildly agree, 3 = unsure, 4 = mildly disagree, 5 = strongly disagree; and thus, with a reverted scale for odd-numbered questions. The NEP reflects either an eco-centered (nature-centered) perspective; a high NEP score identify more closely with concepts that see humans as part of natural systems and emphasize the intrinsic value of nature, or an anthropocentric (human-centered) perspective; a low NEP score identify more closely with concepts that place humans apart from, or above, natural systems and the prioritizing of human needs. The total NEP score for all items is divided by the number of items to give a mean total NEP score (min 1-max 5). The researchers have permission to use the Swedish version of NEP in this study from the rightsholders (43). The item interrelatedness (Cronbach’s *α*) of the measure was 0.727 for the total score.

#### Attitudes toward sustainability and climate change in physiotherapy

The Sustainability Attitudes in Nursing Survey 2 (SANS-2) is a valid and reliable measure developed to assess nurses’ attitudes toward sustainability and climate change (50). SANS-2 broadly asks respondents to indicate whether they agree/disagree with the importance of climate change and sustainability in nursing and their inclusion into nursing education, and therefore, may be applicable to various other groups. The measure has been adapted and used in a nursing faculty setting (43, 51) as well as among paramedic students (52). Further, it was adapted for use in the present study by exchanging the word nursing to physiotherapy. The item interrelatedness (Cronbach’s *α*) of the measure was 0.776 for the total score after changing the word nursing to physiotherapy. It comprises five items assessed on a 7-point Likert scale (1 = strongly disagree to 7 = strongly agree), with higher scores indicating more positive attitudes toward sustainability and climate change (min 1-max 7). The total SANS-2 score for all items is divided by the number of items to give a mean total SANS-2 score (min 1-max 7). The researchers have permission to use the Swedish version of SANS-2 in this study from the rightsholder (53).

#### Education for sustainable development and sustainable healthcare

Fourteen questions on knowledge, attitudes, and self-efficacy related to education for sustainable development and sustainable healthcare are informed by a previously published study, and the researchers have permission to use these questions from the rightsholder (54). One question related to education for sustainable development and the requirement for specific pedagogical approaches is specifically designed for the present study. The 15 questions are divided into three sections (knowledge, attitudes, and self-efficacy) and each question is rated on a Lickert scale (strongly disagree to strongly agree). The item interrelatedness (Cronbach’s *α*) of the measure was 0.897 for knowledge items, 0.748 for attitudes items, 0.884 for self-efficacy items.

## Data analysis

All data were analyzed using descriptive statistics of frequencies, mean, standard deviation, and percentiles. The assumption of a normal distribution was evaluated using the Kolmogorov–Smirnov test. The item interrelatedness of the respective measures was calculated using Cronbach’s *α* which is based on the degree of association between the item responses obtained on a single administration of the measure to a group of people. Cronbach’s α is commonly interpreted using a rules-of-thumb threshold for acceptable interrelatedness of α value of at least 0.70 (55). The Pearson correlation coefficient was calculated for the correlation. The cutoff value for the strength of the correlation used was high (*r* = > ±0.7), moderate (*r* = ±0.5–0.7), low (*r* = ±0.5–0.3), and negligible (*r* = <0.3), and the level of significance was set at *p* = 0.05 (56). The statistical software, SPSS version 28.0.1.1 was used in the analyses.

## Results

### Participants

The survey was completed by a total of 72 educators (76% females, *n* = 55) from undergraduate physiotherapy programs at five higher education institutions in Sweden. However, 50% of the educators came from one of the higher education institutions. The educators were within the age groups 20–30 years (7%, *n* = 5), 31–40 years (15%, *n* = 11), 41–50 years (32%, *n* = 23), 51–60 years (33%, *n* = 24), 61–70 years (11%, *n* = 8), and 71–80 years (1%, *n* = 1). Fifty-three percent, *n* = 38 assigned their primary employment to the university, 33%, *n* = 24 to health care, 1 %, *n* = 1 to shared employment, and 13%, *n* = 9 to another employer. Eleven of the men (64%) and 27 (49%) of the women were employed at a university, and four (23%) of the men and 20 (36%) of the women within health care. The vast majority were physiotherapists by profession (86%, *n* = 62), 4 %, *n* = 3 were either medical doctors, psychologists, or physiologists, and 10%, *n* = 7 had other professions. The larger part of the educators (59%, *n* = 42) had a teaching position, while 29%, *n* = 21 were educators within clinical practice, and 12%, *n* = 9 held another position. Five (29%) of the men and 16 (29%) of the women were educators within clinical practice. The educators were active as educators in years 1, 2 and/or 3 of the education, and their years of experience as an educator varied from 1–5 years up to 31–35 years.

### Consciousness of sustainable development

The educators’ consciousness of sustainable development was high, with 76% reported being aware of Agenda 2030 and the sustainable development goals, and the total mean score of the Sustainability Consciousness Questionnaire for the participants was 4.49 (SD 0.34; Table 1). However, 17%, (*n* = 12) had included, content or perspectives related to sustainable development, either explicitly or implicitly, in their teaching and learning activities during 2022 or 2023, and there were various reasons for not doing so (Table 2).

**Table 1:** Educators’ consciousness of sustainable development (*n* = 72).

Awareness of Agenda 2030 and the global sustainable development goals, Yes/No, n (%)	55/17 (76/24)
Attendance in seminars or courses on sustainable development and/or climate change in the past 12 months, Yes/No, n (%)	23/49 (32/68)
Consciousness of sustainable development, min-max: 1–5, m (SD), *n* = 67*	
Total score of the Sustainability Consciousness Questionnaire	4.49 (0.34)
**Knowingness**	4.36 (0.52)
Economic	4.35 (0.61)
Social	4.61 (0.50)
Environmental	4.11 (0.73)
**Attitudes**	4.74 (0.36)
Economic	4.71 (0.41)
Social	4.78 (0.38)
Environmental	4.73 (0.44)
**Behaviors**	4.36 (0.41)
Economic	3.76 (0.75)
Social	4.77 (0.44)
Environmental	4.55 (0.46)

*5 participants had an incorrect questionnaire completion on the Sustainability Consciousness Scale with 1–3 questions in total with missing values.

**Table 2:** Reasons* for not including content or perspective of sustainable development in teaching and learning activities within undergraduate physiotherapy education (*n* = 72).

Reasons	n, (%)
Not included in the learning outcomes	34 (47%)
Not relevant to my teaching topic	21 (29%)
Do not know *what* to teach	34 (47%)
Lack of knowledge	31 (43%)
Do not know *how* to teach	22 (31%)
No space for it in the course	18 (25%)
Lack of time	18 (25%)
Lack of pedagogical support	8 (11%)
No support from the management	5 (7%)
Do not think students are interested	3 (4%)
Disagree with colleagues	1 (1%)

*Each participant could provide more than one reason.

### Ecological worldview

The total mean score of the New Ecological Paradigm Scale for the participants was 4 (SD 0.41), indicating an eco-centered ecological worldview. The 25th, 50th, and 75th percentiles were 3.8, 4, and 4.33, respectively. Statement 10, ‘The so-called “ecological crisis” facing humankind has been greatly exaggerated’, showed the highest score (mean 4.61). On the contrary, the lowest score (mean 2.49) was attributed to statement 14 ‘The earth has plenty of natural resources if we just learn how to develop them’. The items and responses in the New Ecological Paradigm Scale are summarized in Supplementary material.

### Attitudes toward sustainability and climate change in physiotherapy

The mean total score of the Sustainability Attitudes in Nursing Survey 2 for the participants was 5.49 (SD 0.99), with the 25th, 50th, and 75th percentiles at 4.85, 5.6, and 6.15, respectively, indicating rather positive overall attitudes toward the relevance of the topics of sustainability and climate change within physiotherapy. The larger part of the participants agreed (5–7 on a Lickert scale) that sustainability is an important issue for physiotherapy (86%) and that it should be included in the curriculum (82%). However, the attitudes toward including climate change in the physiotherapy curriculum were less positive, with one-third of the participants (28%) disagreeing (1–3 on a Lickert scale) that it should be included in the curriculum. The items and responses in the Sustainability Attitudes in Nursing Survey 2 are summarized in Table 3.

**Table 3:** Sustainability and climate change attitudes among physiotherapy educators (*n* = 72).

SANS* items	Strongly agree 7	6	5	4	3	2	Strongly disagree 1	
	N (%)	n (%)	n (%)	n (%)	N (%)	n (%)	n (%)	Mean (SD)
1. Climate change is an important issue for physiotherapy.	20 (27.8)	10 (13.9)	20 (27.8)	12 (16.7)	7 (9.7)	2 (2.8)	1 (1.4)	5.19 (1.51)
2. Issues about climate change should be included in the physiotherapy curriculum.	12 (16.7)	9 (12.5)	18 (25)	13 (18.1)	13 (18.1)	5 (6.9)	2 (2.8)	4.60 (1.62)
3. Sustainability is an important issue for physiotherapy.	31 (43.1)	17 (23.6)	14 19.4	7 (9.7)	3 (4.2)	0	0	5.92 (1.18)
4. Sustainability should be included in the physiotherapy curriculum.	27 (37.5)	15 (20.8)	17 (23.6)	7 (9.7)	3 (4.2)	1 (1.4)	2 (2.8)	5.63 (1.48)
5. I apply sustainability principles at home.	30 (41.7)	23 (31.9)	15 (20.8)	4 (5.6)	0	0	0	6.10 (0.92)

*The Sustainability Attitudes in Nursing Survey 2.

### Correlation between ecological worldview and attitude toward sustainability and climate change within physiotherapy

The correlation between ecological worldview (New Ecological Paradigm Scale) and attitudes toward sustainability and climate change (Sustainability Attitudes in Nursing Survey 2) was low (*r* = 0.356) and statistically significant (*p* < 0.002).

### Education for sustainable development and sustainable healthcare

The larger part of the participants agreed or strongly agreed, (81%) about having content knowledge on climate and health, and half of the participants agreed or strongly agreed, (54%) about having content knowledge on sustainable health care. A smaller part of the participants agreed or strongly agreed, (17–25%) about having pedagogical content knowledge on education for sustainable development (items 3–5). A moderate to large part of the participants (56–88%) agreed or strongly agreed with the significance of health professionals and healthcare for sustainable development (items 6–10). Approximately half of the educators (51%) were unsure whether education for sustainable development requires specific teaching approaches. There was a wide variation in how participants perceived their self-efficacy in education for sustainable development and education for sustainable healthcare. The items and responses are summarized in Table 4.

**Table 4:** Physiotherapy educators self-reported knowledge, attitudes, and self-efficacy related to education for sustainable development and education for sustainable healthcare (*n* = 72).

	Strongly agree n (%)	Agree n (%)	Unsure n (%)	Disagree n (%)	Strongly disagree n (%)
Knowledge					
1. I can list a few examples of direct effects of climate change on human health	27 (37.5)	31 (43.1)	11 (15.3)	3 (4.2)	0
2. I know what healthcare professionals in my field can do to take action to reduce negative environmental impact	4 (5.6)	35 (48.6)	23 (31.9)	5 (6.9)	5 (6.9)
3. I know how to best explain what sustainable development means for healthcare professionals	5 (6.9)	13 (18.1)	31 (43.1)	14 (19.4)	9 (12.5)
4. I am aware of how to best teach my students about the importance of sustainable development issues for their future work	3 (4.2)	9 (12.5)	31 (43.1)	18 (25)	11 (15.3)
5. I know how best to inspire my students to be interested in sustainable development issues	3 (4.2)	15 (20.8)	27 (37.5)	14 (19.4)	13 (18.1)
Attitudes					
6. The healthcare sector is part of the sustainability problem	29 (40.3)	29 (40.3)	11 (15.3)	3 (4.2)	0
7. Sustainable development should be the core business for healthcare professionals	11 (15.3)	29 (40.3)	14 (19.4)	14 (19.4)	4 (5.6)
8. Healthcare professionals have a critical role to play in achieving sustainability	9 (12.5)	32 (44.4)	24 (33.3)	5 (6.9)	2 (2.8)
9. Healthcare professionals should be equipped to advocate for change for sustainability	29 (40.3)	32 (44.4)	6 (8.3)	4 (5.6)	1 (1.4)
10. All health professions students should learn about sustainable development	34 (47.2)	29 (40.3)	4 (5.6)	4 (5.6)	1 (1.4)
11. Education for sustainable development requires specific pedagogical approaches	2 (2.8)	15 (20.8)	37 (51.4)	14 (13.9)	11 (11.1)
Self-efficacy					
12. I am confident in expanding my students´ awareness of the concept of sustainable development	7 (9.7)	28 (38.9)	25 (34.7)	8 (11.1)	4 (5.6)
13. I am confident in explaining the relationship between sustainable development and my teaching content	3 (4.2)	21 (29.2)	36 (50)	9 (12.5)	3 (4.2)
14. I am confident in teaching how the healthcare sector affects the environment	6 (8.3)	24 (33.3)	28 (38.9)	3 (4.2)	11 (15.3)
15. I am confident in teaching effective ways of implementing sustainable development practices at work	5 (6.9)	18 (25)	32 (44.4)	8 (11.1)	9 (12.5)

## Discussion

To our knowledge, this is the first study to describe physiotherapy educators’ overall consciousness of sustainable development, ecological worldviews, attitudes toward sustainability and climate change in physiotherapy, as well as views on education for sustainable development and sustainable healthcare in physiotherapy undergraduate education. Our findings indicate an overall high consciousness but a rather low application of sustainable development in teaching and learning among educators in Sweden. An interesting finding was that although the educators demonstrated a rather high endorsement of eco-centric ecological worldviews and fairly positive attitudes toward sustainability in physiotherapy, attitudes toward including climate change in the physiotherapy curriculum were less positive. The findings also indicated a low correlation between educators’ ecological worldviews and their overall attitudes toward sustainability and climate change in physiotherapy. Furthermore, less than 20 percent of the educators had included content or perspectives related to sustainable development in their teaching and learning activities.

The educators in the present study demonstrated somewhat stronger eco-centered ecological worldviews than the general population in Sweden and other high-income countries (57, 58). It was also slightly stronger than among nurses and physicians in Sweden (36) but somewhat weaker than among environmentalists in the United Kingdom and Norway (58, 59). The findings of less positive attitudes toward including climate change in physiotherapy education give rise to a question of whether or not educators endorse an eco-centric perspective privately but adopt a more anthropocentric perspective in their professional roles. While there is limited research on this phenomenon within the framework of the New Ecological Paradigm Scale, previous research indicates that healthcare professionals take more action in their personal than professional lives to protect the environment, particularly those with strong professional identities (60). This aligns with findings that pro-environmental behavior does not easily transfer between personal and professional contexts (61, 62). However, difficulties implementing issues related to sustainable development and climate change at work within healthcare or higher education might also be due to work culture, established policies, or attitudes of the staff or leadership (63). There is also growing recognition that the traditional model of sustainable development may be insufficient to address the scale and complexity of the ecological crises (64–66). The eco-centric worldview that most of the educators in this study hold, challenges the anthropocentric assumptions often embedded within the discourse on sustainable development, where environmental actions are sometimes justified solely as means to ensure human survival rather than as ethical imperatives to protect the inherent worth of the natural world.

In healthcare, the focus is often on immediate patient needs, with professional identity often tied to an anthropocentric perspective, where human individual health is the primary concern. Consistent with previous research (13, 41, 67), educators within physiotherapy in Sweden understood the effects of climate change on human health and that healthcare is part of the sustainability problem. However, increasing emphasis is also being placed on the critical role of health professionals as leaders, educators, and advocates in addressing environmental deterioration such as climate change (39, 68–71). To address this, frameworks like sustainable healthcare practices (20) and learning outcomes for sustainable healthcare (21) need to be integrated into physiotherapy education, practice, and research in Sweden (26, 72). Making sustainable healthcare “business as usual” in healthcare, with organizational support for climate-resilient services (73), might be crucial for fostering future physiotherapists equipped to navigate this transition. As noted by the Swedish National Council on Medical Ethics (74), healthcare professionals must contribute to climate action due to its profound public health impact. Educators must, therefore, embrace moral courage, speak up when ethical values are compromised (75) by reinterpreting the principles of primum non nocere (first, do no harm) and recognizing the vitality of the planet as the fundamental foundation for human health (76). World Physiotherapy’s Position Statement for Climate and Health provides a pathway to promote and incorporate environmental sustainability into practice (77). The statement emphasizes the importance of physiotherapists acknowledging and addressing the health consequences of climate change, which highlights the growing expectation for higher education to prepare healthcare professionals for sustainability challenges.

The less positive attitude toward integrating climate change in physiotherapy education may also partly stem from political discourse, which can influence how it is approached in professional contexts. In Sweden, where climate activism is highly visible, eco-centric ecological worldviews may be privately supported, but there may also be perceptions that discussing climate change in education is risky or inappropriate, especially if seen as controversial (78). A large global survey of health professionals showed that 16% were hesitant to communicate about climate change due to concerns about controversy and 14% due to professional risk (41). How this dynamic plays out in Sweden’s higher education remains unclear, but by framing education for sustainable development and sustainable healthcare through the lens of health consequences (79, 80), educators can create space for these discussions without falling into political traps. Leadership from national organizations like the Swedish Association of Physiotherapists (81) might play a pivotal role in reshaping professional norms within physiotherapy. By integrating sustainable development within planetary boundaries into the profession’s core identity, physiotherapists can be positioned as key actors in addressing both human and planetary health. The Swedish Society of Medicine and the Swedish Society of Nursing have already set guidelines for their respective professions to adopt sustainable and climate-conscious healthcare practices (82, 83). This momentum should inspire the physiotherapy profession in Sweden to similarly advance and keep pace with this transition.

Nearly half of the educators who did not teach for sustainable development referred to the absence of relevant learning outcomes or uncertainty about what to teach. While they rated their content knowledge of sustainable development as relatively high, they were unsure about how to teach or inspire students on this topic. When educators are tasked with a teaching and learning topic in which they might not have been formally trained or have limited practical experience, it can pose significant challenges. This can lead to ambiguity regarding the purpose of learning, resulting in a focus on learning *about* sustainability rather than a clear emphasis on learning *for* sustainability, even if its exact meaning within the field remains emergent. This situation might necessitate a transformation not only for the educator but also for the education itself (84). What is meant by development in physiotherapy? what should be learned and unlearned within physiotherapy? and how can physiotherapists contribute to shaping peaceful, just, and sustainable futures? According to Sterling (85), education should not be confined to “doing things better” or simply “doing better things.” Instead, it should aim for a deeper level of change, known as transformative learning. This approach encourages a fundamental shift in perspective, enabling individuals to see and understand the world, for example, the role of physiotherapy, in entirely new ways (86). The educators’ uncertainty may also reflect the challenge of applying all dimensions of sustainable development or sustainable healthcare in educational practice. Cotton et al. found substantial variation in how sustainability was understood by lecturers from different disciplines within higher education, with a notable tendency to focus more on environmental issues than on social and economic dimensions of sustainability (87). This could also be valid for physiotherapy educators in Sweden, who might use a single dimension of sustainable development in their teaching and learning activities or unconsciously educate about or for sustainable development without explicitly using the framework. This study explored educators’ consciousness, attitudes, and knowledge of sustainable development and sustainability without providing a specific model or definition to the educators. However, a definition of sustainable healthcare was provided: “Sustainability in healthcare means designing and providing care that uses resources in a way that does not negatively affect future health and well-being.” Thus, educators could have conceptualized sustainable development without any framework or with different sustainability lenses such as through weak sustainability approaches, i.e., the three-pillar model (88), or Venn diagram (89), or a stronger sustainability approach, i.e., the nested dependency model (4). Education for sustainable development has been criticized for its perceived vagueness and the challenge of integrating its principles across educational contexts (90–92). The broad and often abstract nature of sustainable development can lead to superficial integration within curricula, where the focus may be more on rhetoric than on substantial changes in educational practices. Thus, adopting a vision for sustainable development in physiotherapy practice in Sweden involves not only paradigmatic changes in education but also in the operational and policy frameworks guiding healthcare delivery, since part of undergraduate physiotherapy education also occurs within clinical practice. In 2018 a number of Swedish key stakeholders in local authorities and regions described major variations in how they viewed the meaning of the concept of sustainable development and its implications for the work of healthcare. Few used the term sustainable healthcare and none mentioned an environmental dimension of sustainability (93). Undergraduate physiotherapy education and educators can thus play a pivotal role by broadening curricula and the understanding of the concept by implementing sustainable development rooted in a variety of cultural traditions, including ecological Indigenous health knowledge (94). This would foster a new generation of physiotherapists who might appreciate and use alternative health knowledge systems when creating future health solutions (95).

Educators were also uncertain if specific teaching methods were needed for education for sustainable development, perhaps reflecting a tendency in academia to discuss issues based on one’s own conceptual understanding and not based on practical application. Similar gaps have been identified in nursing education, where environmental issues are treated conceptually rather than with hands-on engagement (96). Previous research on education for sustainable healthcare has also shown that it often has single-session designs and is primarily theory-based where implication for clinical practice is lacking (40). However, several pedagogical approaches can effectively foster sustainability competencies with a focus on action competence (97, 98). There are also numerous resources and frameworks to facilitate the implementation of sustainable development and sustainable healthcare into curricula across health professions (99–101). The concept of planetary health offers a meaningful framework for operationalizing an eco-centric worldview within education (102). Such an approach extends beyond the traditional pillars of sustainable development, emphasizing the need for transformative change in societal values and behaviors to realign human activities within the Earth’s ecological limits. Revising or adding learning outcomes at an overall local program level could also provide clearer direction for involved educators at the course level. This includes strategic and role-modeling leadership that engages and empowers educators to drive the development of education for sustainable development and to take responsibility for acting as stewards of the planet (103). Thus, it seems as if there is a strong need for continuous professional development among educators within physiotherapy in Sweden to prepare future physiotherapists for evolving environmental and healthcare challenges. A national discussion on how physiotherapy in Sweden should embrace sustainable development as well as education for sustainable development and sustainable healthcare is also essential. This could facilitate the transition toward a more comprehensive paradigm that also embraces the educators’ eco-centered ecological worldview. Such a discussion can also draw attention to the challenges of sustainable development as a concept that can be selected, misunderstood, and misplaced in education due to ambiguity, with the consequence that the topic can be perceived as meaningless, reducing its potential to create change. Further exploration of educators’ perceptions of education for sustainable development in physiotherapy would also provide valuable insights into how teaching and learning activities may be organized to support students learning for sustainable development within planetary boundaries.

Some strengths and limitations of the present study must be acknowledged. The survey was based on self-report, which may have resulted in socially desired answers. This is an inherent limitation in most surveys and while it was ensured the data collection was anonymous, self-reports are never free from bias (104). It is also possible that the study attracted those already interested in sustainable development, and that educators who responded to the survey differed from those who did not, which may have resulted in selection bias. However, the inclusion of educators from both campus and health care and other professional backgrounds than physiotherapy strengthens the findings’ transferability to educators both within clinical practice and interdisciplinary educators alike. It should also be noted that the overall response rate could not be calculated, as the exact numbers of educators within the undergraduate physiotherapy education could not be retrieved. Further, the measures included in the survey did not answer the educators’ positionality on sustainable development, i.e., preferred definition or if a weak or strong sustainability approach was used when answering the survey. The inclusion of different concepts in the survey, such as Agenda 2030, the sustainable development goals, sustainable development, and sustainable healthcare may also have contributed to ambiguities for the educators. The Sustainability Attitudes in Nursing Survey 2 only covers one part of the triple planetary crisis (105), namely climate change, but not pollution and loss of biodiversity. However, at the time of the development of the Sustainability Attitudes in Nursing Survey 2 (50), this concept was not popularized and officially introduced to the healthcare community. The Swedish versions of the New Ecological Paradigm Scale and Sustainability Attitudes in Nursing Survey 2, as well as the questions on education for sustainable development and sustainable healthcare, had not been validated in physiotherapy settings prior to the use in this study, representing an additional limitation of this study. However, they have been used in several studies in other healthcare contexts with satisfying results. Further, even though the item interrelatedness for all measures was within the commonly used rule-of-thumb for acceptable value, the use of this cut-off value has been criticized for the risk of the researcher’s efforts to increase alpha above a certain level by adding or deleting items (106). However, all items in the Sustainability Attitudes in Nursing Survey 2, the New Ecological Paradigm Scale, and the Sustainability Consciousness Questionnaire were used as in the original versions of the measures. One question was added to the previously published questions on education for sustainable development and sustainable healthcare, which in fact decreased the Cronbach’s *α* value within attitudes.

## Conclusion

Despite the endorsement of eco-centered ecological worldviews and a rather high consciousness of sustainable development as an overall concept, there remains a disconnect between educational attitudes and actions among Swedish physiotherapy educators. This points to the need to explore the narrative of sustainable development within physiotherapy in Sweden rooted in broader concept understanding, ethics, and reflective practice for sustainable development. A key priority should be to offer new perspectives on professional identity and continuing professional development within sustainable development. Educators might need support in translating their general consciousness into valuable applications within their professional roles.

## Data Availability

The raw data supporting the conclusions of this article will be made available by the authors, without undue reservation.
