# Supplementary material for: High consciousness—low application: sustainable development and sustainable healthcare in undergraduate physiotherapy education in Sweden
Source: Front Public Health. 2024 Dec 16;12:1509997. doi: 10.3389/fpubh.2024.1509997 (PMC11685746; doi:10.3389/fpubh.2024.1509997)
Supplement: Supplementary file 1 [file Table_1.docx]

**Supplementary material.** New Environmental Paradigm (NEP) scale among physiotherapy educators (n=72). * Agreement to the eight odd-numbered items and disagreement to the seven even-numbered items indicate pro-environmental responses = score 1-5)

| NEP items | Strongly agree  1 or 5 * | Mildly agree  2 or 4 * | Unsure  3 * | Mildly disagree  4 or 2 * | Strongly disagree  5 or 1 * | Mean  (SD) |
| --- | --- | --- | --- | --- | --- | --- |
|  | N  (%) | n  (%) | n  (%) | n  (%) | n  (%) |  |
| 1. We are approaching the limit of the number of people the earth can support. | 25  (35.2) | 19  (26.8) | 18  (25.4) | 6  (8.5) | 3  (4.2) | 3.80  (1.14) |
| 2. Humans have the right to modify the natural environment to suit their needs. | 1  (1.4) | 22  (30.6) | 8  (11.1) | 31  (43.1) | 10  (13.9) | 3.38  (1.11) |
| 3. When humans interfere with nature it often produces disastrous consequences. | 22  (30.6) | 45  (62.5) | 2  (2.8) | 3  (4.2) | - | 4.19  (0.69) |
| 4. Human ingenuity will insure that we do NOT make the earth unlivable. | 2  (2.8) | 10  (13.9) | 21  (29.2) | 27  (37.5) | 12  (16.7) | 3.51  (1.02) |
| 5. Humans are severely abusing the environment. | 50  (69.4) | 17  (23.6) | 3  (4.2) | 2  (2.8) | - | 4.60  (0.71) |
| 6. The earth has plenty of natural resources if we just learn how to develop them. | 12  (16.7) | 32  (44.4) | 13  (18.1) | 11  (15.3) | 4  (5.6) | 2.49  (1.11) |
| 7. Plants and animals have as much right as humans to exist. | 48  (66.7) | 18  (25) | 1  (1.4) | 5  (6.9) | - | 4.51  (0.84) |
| 8. The balance of nature is strong enough to cope with the impacts of modern industrial nations. | - | 3  (4.2) | 5  (6.9) | 24  (33.3) | 40  (55.6) | 4.40  (0.80) |
| 9. Despite our special abilities humans are still subject to the laws of nature. | 34  (47.2) | 30  (41.7) | 7  (9.7) | 1  (1.4) | - | 4.35  (0.72) |
| 10. The so-called “ecological crisis” facing humankind has been greatly exaggerated. | - | 2  (2.8) | 3  (4.2) | 16  (22.2) | 51  (70.8) | 4.61  (0.70) |
| 11. The earth is like a spaceship with very limited room and resources. | 12  (16.7) | 35  (48.6) | 16  (22.2) | 6  (8.3) | 3  (4.2) | 3.65  (1.0) |
| 12. Humans were meant to rule over the rest of nature. | - | 2  (2.8) | 6  (8.3) | 24  (33.3) | 40  (55.6) | 4.42  (0.77) |
| 13. The balance of nature is very delicate and easily upset. | 33  (45.8) | 28  (38.9) | 4  (5.6) | 6  (8.3) | 1  (1.4) | 4.19  (0.97) |
| 14. Humans will eventually learn enough about how nature works to be able to control it. | 1  (1.4) | 11  (15.3) | 23  (31.9) | 28  (38.9) | 9  (12.5) | 3.46  (0.95) |
| 15. If things continue on their present course, we will soon experience a major ecological catastrophe. | 43  (59.7) | 23  (31.9) | 3  (4.2) | 3  (4.2) | - | 4.47  (0.77) |
